# ERas regulates cell proliferation and epithelial–mesenchymal transition by affecting Erk/Akt signaling pathway in pancreatic cancer

**DOI:** 10.1007/s13577-020-00401-2

**Published:** 2020-07-22

**Authors:** Yang Liu, Peng Qin, Rong Wu, Lianfang Du, Fan Li

**Affiliations:** 1 Department of Ultrasound, Shanghai General Hospital, Shanghai Jiaotong University School of Medicine, Shanghai, 200080 China; 2 Department of Instrument Science and Engineering, Shanghai Jiao Tong University, Shanghai, 200240 China

**Keywords:** ERas, siRNA, Erk/Akt, Epithelial–mesenchymal transition (EMT), Pancreatic cancer

## Abstract

Pancreatic cancer is the fourth most common lethal malignancy with an overall 5-year survival rate of less than 5%. ERas, a novel Ras family member, was first identified in murine embryonic stem cells and is upregulated in various cancers. However, the expression and potential role of ERas in pancreatic cancer have not been investigated. In this study, we found that ERas mRNA and protein were upregulated in pancreatic cancer tissues and cells compared with controls. Knockdown of ERas in pancreatic cancer cells by siRNA significantly decreased cell proliferation, colony formation, migration, and invasion and promoted cell apoptosis in vitro. Epithelial–mesenchymal transition (EMT) is closely related to tumor progression. We observed a significant decrease in N-cadherin expression in pancreatic cancer cells in response to ERas gene silencing by immunofluorescence assay and western blot. Furthermore, tumor growth and EMT were inhibited in xenografts derived from pancreatic cancer cells with ERas downregulation. We further investigated the regulatory mechanisms of ERas in pancreatic cancer and found that ERas may activate the Erk/Akt signaling pathway. Moreover, Erk inhibitor decreased pancreatic cancer cells proliferation and colony formation activities. Our data suggest that targeting ERas and its relevant signaling pathways might represent a novel therapeutic approach for the treatment of pancreatic cancer.

**Electronic supplementary material:**

The online version of this article (10.1007/s13577-020-00401-2) contains supplementary material, which is available to authorized users.

## Introduction

Pancreatic cancer is the fourth leading cause of cancer-related mortalities with rapid progression and dismal prognosis [1–5]. The median survival rate of pancreatic cancer is less than 6 months [6–8]. Chemotherapy plays an important role in the treatment of pancreatic cancer. However, pancreatic cancer can show intrinsic resistance to chemotherapy, resulting in aggressive local invasion and early metastasis of pancreatic cancer cells (PCCs) [9,10]. Thus, there is a desperate need for innovative treatment strategies to improve the prognosis of patients with pancreatic cancer [11].

Embryonic stem (ES) cells are pluripotent cells that maintain differentiation ability [12]. Due to their rapid growth and immortality, ES cells have been used in stem cell therapies. However, ES cells will produce teratomas within several weeks after transplantation, limiting their therapeutic usage [13]^.^ Thus, ES cells and tumor cells most likely share common growth properties.

ERas is a new member of the Ras family and shares high identity to conventional Ras oncogenes (Hras, Kras and Nras). The gene encoding ERas, located at chromosome Xp11.23, was first found in murine ES cells [14] and initially identified as a pseudogene [15,16]. Subsequent studies showed that ERas plays an important function in the survival of murine ES cells [17–19]. Recent studies have demonstrated that ERas plays a critical role in the occurrence and progression of many malignant tumors, such as gastric cancer [20], breast cancer [21], and neuroblastoma [22].

In this study, we not only identified the expression of ERas in pancreatic cancer cells, but also elucidated the role and potential mechanisms of ERas in pancreatic cancer. Meanwhile, we assumed that Erk/Akt pathway helped the development of PaC which might provide a potential oncogenic mechanism of ERas in PaC. Therefore, early therapy targeting Akt and Erk pathways might produce an inhibitory effect on PaC and reduce the chemotherapy resistance. Accordingly, we believe ERas might be an extremely key factor participated in cell differentiation and movement via Erk and Akt signaling pathway.

## Materials and methods

### Experimental animals

This study was performed in strict accordance with institutional guidelines and approved by the Institutional Animal Care and Use Committee (IACUC) of the Shanghai Model Organisms Center (permit number 2019-0011). Animals used in this experiment were purchased from Shanghai Slack Laboratory Animals Co., Ltd (China).

### Cell lines and reagents

Human PCC lines BxPC3, Capan-1, CFPAC-1, Panc-1 and SW1990 as well as the normal human pancreatic duct epithelial (HPDE) cell line were obtained from the Type Culture Collection of the Chinese Academy of Sciences. Fetal bovine serum (FBS) was purchased from Gibco BRL (Gaithersburg, MD, USA), and Dulbecco's modified Eagle's medium (DMEM) and RPMI 1640 were obtained from Hyclone (Logan, UT, USA). HPDE, Panc-1 and Capan-1 cells were maintained in DMEM with 10% FBS. BxPC3 and SW1990 cell lines were maintained in RPMI 1640 with 10% FBS. CFPAC-1 cells were maintained in Iscove’s Modified Dulbecco's Medium (IMDM) with 10% FBS. All cells were cultured according to standard protocol. The Erk inhibitor SCH772984 was obtained from Selleck Chemicals (Houston, TX, USA) and dissolved in 100% dimethyl sulfoxide (DMSO) at a concentration of 10 mM.

### Small interfering RNA transfection

Two ERas stealth siRNAs (siRNA30 and siRNA32) and the control siRNA were designed and synthesized by Bioneer (Daejon, Korea) (Table 1). siRNAs were mixed with Lipofectamine™ 2000 in Opti-MEM™ reduced serum medium (31985070, Gibco BRL, Gaithersburg, Md) for 5 min at room temperature and then added to each well of a 24-well plate containing SW1990 or Panc-1 cells. The cells were harvested for subsequent experiments after 24 h of transfection.

**Table 1:** The sequences of ERas siRNA

Name	Sequences
siRNA 30	F: GCAACUAGCUUUGAGGGAC(dTdT) R: GUCCCUCAAAGCUAGUUGC(dTdT)
siRNA 32	F: GUAACAUGGGAGUGCCUAA(dTdT) R: UUAGGCACUCCCAUGUUAC(dTdT)
Negative control siRNA	F: CCUACGCCACCAAUUUCGU(dTdT) R: ACGAAAUUGGUGGCGUAGG (dTdT)

### RNA isolation and quantitative real-time PCR (qRT-PCR)

Total RNA was extracted from cells and tissue samples with Trizol. Reverse transcription was performed using the Takara Reverse Transcription Kit according to the manufacturer’s instructions, followed by real-time quantitative PCR using the Takara SYBR Premix ExTaq kit, according to the manufacturer’s instructions. The primers are shown in Table 2.

**Table 2:** The sequences of primers

Name	Sequences
GAPDH	F: 5ʹ-ggacctgacctgccgtctag-3ʹ R: 5ʹ-gtagcccaggatgcccttga-3ʹ
ERas	F: 5ʹ-ggacctgacctgccgtctag-3ʹ R: 5ʹ-gtagcccaggatgcccttga-3ʹ

### Western blot analysis

Cells were lysed with RIPA lysis buffer and phenylmethanesulfonyl fluoride (100:1), and protein concentration was determined using the BCA Protein Assay Kit (#23227, Pierce, USA). Protein samples (50 μg) were loaded onto acrylamide gels for electrophoresis and then transferred onto polyvinylidene fluoride membranes. The membranes were then blocked with 5% non-fat milk for 1 h at room temperature, followed by incubation with primary antibody overnight at 4 °C. Membranes were then incubated with secondary antibodies for 1 h at room temperature and the signals were detected by an enhanced chemiluminescence detection system (Amersham Bioscience, Piscataway, NJ, USA). β-actin was used as the internal control.

The primary antibodies were anti-ERas antibody (1:1000; Abgent), anti-E-cadherin antibody (1:1000; #14472; Cell Signaling Technology), anti-N-cadherin antibody (1:1000; #13116; Cell Signaling Technology), anti-Erk (1:1000; #4695; Cell Signaling Technology), anti-phospho-Erk^Thr202/Tyr204^ (1:1000; #9101; Cell Signaling Technology), anti-Akt (1:1000; #4691; Cell Signaling Technology), anti-phospho-Akt^Ser473^ (1:1000; #4060; Cell Signaling Technology), and rabbit anti-β-actin (1:5000; Abcam). Secondary antibodies included goat anti-rabbit IgG horseradish peroxidase-conjugated secondary antibody (1:2000; #7074; Cell Signaling Technology) or horse anti-mouse IgG horseradish peroxidase-linked secondary antibody (1:2,000; #7076; Cell Signaling Technology).

### Cell proliferation assays

Cell proliferation was assessed by CCK-8 assay and colony formation assay. For CCK-8 assay, siRNA-transfected SW1990 and Panc-1 cells were seeded into 96-well plates (3 × 10^3^ cells/well). After 0, 24, 48, 72, and 96 h, the absorbance was measured using the Cell Counting Kit (Dojindo, Tokyo, Japan) according to the manufacturer’s protocol. For the colony formation assay, siRNA-transfected SW1990 and Panc-1 cells were seeded in six-well plates (200 cells/well) and cultured for 14 days. Colonies were fixed with 1 ml 4% paraformaldehyde containing 0.04% crystal violet for 20 min. The supernatant was discarded and cells were washed with 1 ml ddH_2_O. Images were obtained in five random fields and colonies were counted by ImageJ software. Each experiment was replicated with five independent wells.

### Apoptosis assay

Flow cytometry was used to determine the percentage of apoptotic cells. Cells were double-labeled with Annexin V (AV)-fluorescein isothiocyanate (FITC) and propidium iodide (PI). Cells were washed twice and adjusted to a concentration of 1 × 10^6^ cells/mL with cold PBS. Binding buffer (195 μl), AV-FITC (5 μl) and PI (10 μl) were added to 100 μL of cell suspension and samples were incubated for 20 min at room temperature in the dark. Samples were examined on a Beckman Coulter Flow Cytometer. The data were analyzed by FlowJo 10.

### Hoechst 33342 assay

Hoechst 33,342 staining was used to evaluate cell apoptosis. Cells transfected with siRNA were incubated with Hoechst 33342 (Beyotime, Nantong, China) at 37 °C with 5% CO_2_ for 20 min according to the manufacturer’s protocol. Apoptosis was then assessed by fluorescence microscopy based on the presence of condensed chromatin and micronucleation.

### Cell migration and invasion assay

Cell migration and invasion abilities were assessed by Transwell assay (Corning, California, USA) according to the manufacturer’s instructions. In migration assays, siRNA-transfected SW1990 (3 × 10^4^) and Panc-1 cells (3 × 10^4^) were resuspended in 200 μl serum-free medium and seeded into the upper chamber of the Transwell system. In the invasion assay, Matrigel (BD Biosciences) was included to simulate the extracellular matrix; siRNA-transfected SW1990 (5 × 10^4^) and Panc-1 cells (5 × 10^4^) resuspended in 200 μl serum-free medium were seeded into the upper chamber pre-coated with Matrigel. In both migration and invasion assays, the lower chambers were filled with 500 μl of medium containing 20% FBS as the chemoattractant. Cells were incubated for another 48 h at 37 °C with 5% CO_2_ and then the non-migrating cells on the upper surface of the chamber were gently scraped off. The cells were fixed with 4% paraformaldehyde containing 0.04% crystal violet for 20 min. Migrated or invaded cells were counted under a microscope in at least three random fields.

### Wound-healing assay

SW1990, siRNA-transfected SW1990, Panc-1 and siRNA-transfected Panc-1 cells were seeded into six-well plates and cultured until they achieved 90% confluence. The cell monolayers were then scratched with 200 μl pipette tips. Cells were photographed at 0 and 24 h using an inverted microscope, and the wound width was measured by Image J software. Each analysis was repeated five times.

### Colony formation assay

A total of 200 siRNA-transfected SW1990 or SW1990 cells incubated with DMSO and Erk inhibitor (SCH772984) were plated in six-well plates for 2 weeks. Cell colonies were fixed with 4% methanol for 20 min and stained with 0.04% crystal violet for 20 min. After washing with PBS for 10 min and air drying, colonies were photographed in five random fields and counted by ImageJ software.

### Immunofluorescence assay

EMT, both in vitro and in vivo, was analyzed by immunofluorescence. SW1990 cells were fixed in 4% paraformaldehyde for 20 min and then permeabilized with 0.1% Triton X-100 for 15 min and then incubated with 5% bovine serum albumin (BSA) for 30 min. While in vivo analysis, dewaxed and hydrated mice tumor sections were heated to 95–100 °C in sodium citrate solutions for 30 min in order to repair the antigens. Afterward, sections were also incubated with 5% BSA at room temperature. Both cell samples and tumor sections were incubated with anti-E-cadherin antibody and anti-N-cadherin antibody (1:100) overnight at 4 °C and then incubated with FITC-conjugated goat anti-mouse/rabbit secondary antibodies (1:2000; #SA00003-1/#SA00003-2; Proteintech) at room temperature for 1 h. An Olympus BX-43 microscope was used to capture images.

### Tumor xenograft model and tumorigenicity assay

Twelve 4-week-old male nude mice (weight, approximately 20 g) were housed under specific pathogen-free conditions. Mice were randomly divided into three groups (control, siRNA30 and siRNA32 groups; *n* = 4 mice/group). siRNA-transfected SW1990 cells (3 × 10^6^ cells in 100 μl of PBS) were subcutaneously implanted into the back of mice. The tumor nodules were examined weekly with a caliper. Tumor volume was calculated by the following formula: volume = 0.5 × length × width^2^. Tumor growth curves were calculated. The nude mice were euthanized when the volume of tumors reached 1 cm^3^ in size (after approximately 4 weeks). Tumors were harvested, dissected, weighed and embedded in paraffin or fixed in 10% formalin.

### Immunohistochemistry

Tissues (4 μm thick) were stained with hematoxylin and eosin (H&E). The FITC Rabbit anti-Ki-67 antibody (1:100, 12075, CST) was used to detect Ki-67 expression (nuclear staining), and the Ki-67 index was determined as the percentage of Ki-67-positive cells in 100 cells. The TUNEL assay (Roche, Germany) was performed to detect apoptotic cells in tumor tissues. An Olympus BX-43 microscope was used to capture images.

### Statistical analysis

Statistical analyses were performed using SPSS 16.0 software. Statistical comparisons were conducted by Student’s *t* test and the results are presented as mean ± standard deviation (SD) from at least three separate experiments. A *P *value of 0.05 or less was considered statistically significant.

## Results

### ERas expression is significantly upregulated in pancreatic cancer

**Fig. 1:**
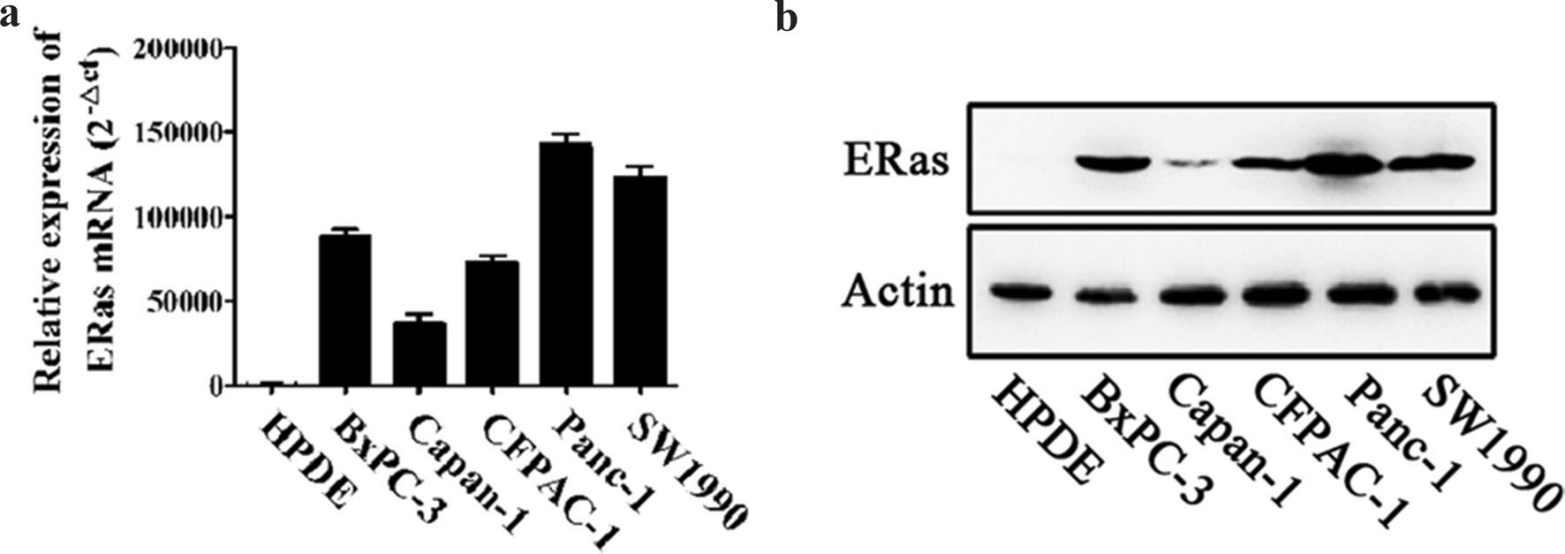
ERas mRNA and protein expression in PCCs. **a** Relative ERas gene expression was examined in five pancreatic cancer cell lines and the normal human pancreatic duct epithelial cell HPDE by real-time PCR. **b** Expression of ERas protein in the indicated cell lines was determined by western blotting

To determine whether ERas is involved in the development of pancreatic cancer, we first analyzed ERas mRNA and protein expression in HPDE and pancreatic cancer cell lines by real-time PCR and western blot analysis. Both ERas mRNA and protein expression were detected in all five pancreatic cancer cell lines, while no expression was detected in the HPDE cell line (Fig. 1a, b). Taken together, these results showed that ERas mRNA and protein expression are significantly upregulated in PCCs.

### Inhibition of ERas expression decreased the proliferation and colony formation abilities of PCCs in vitro

**Fig. 2:**
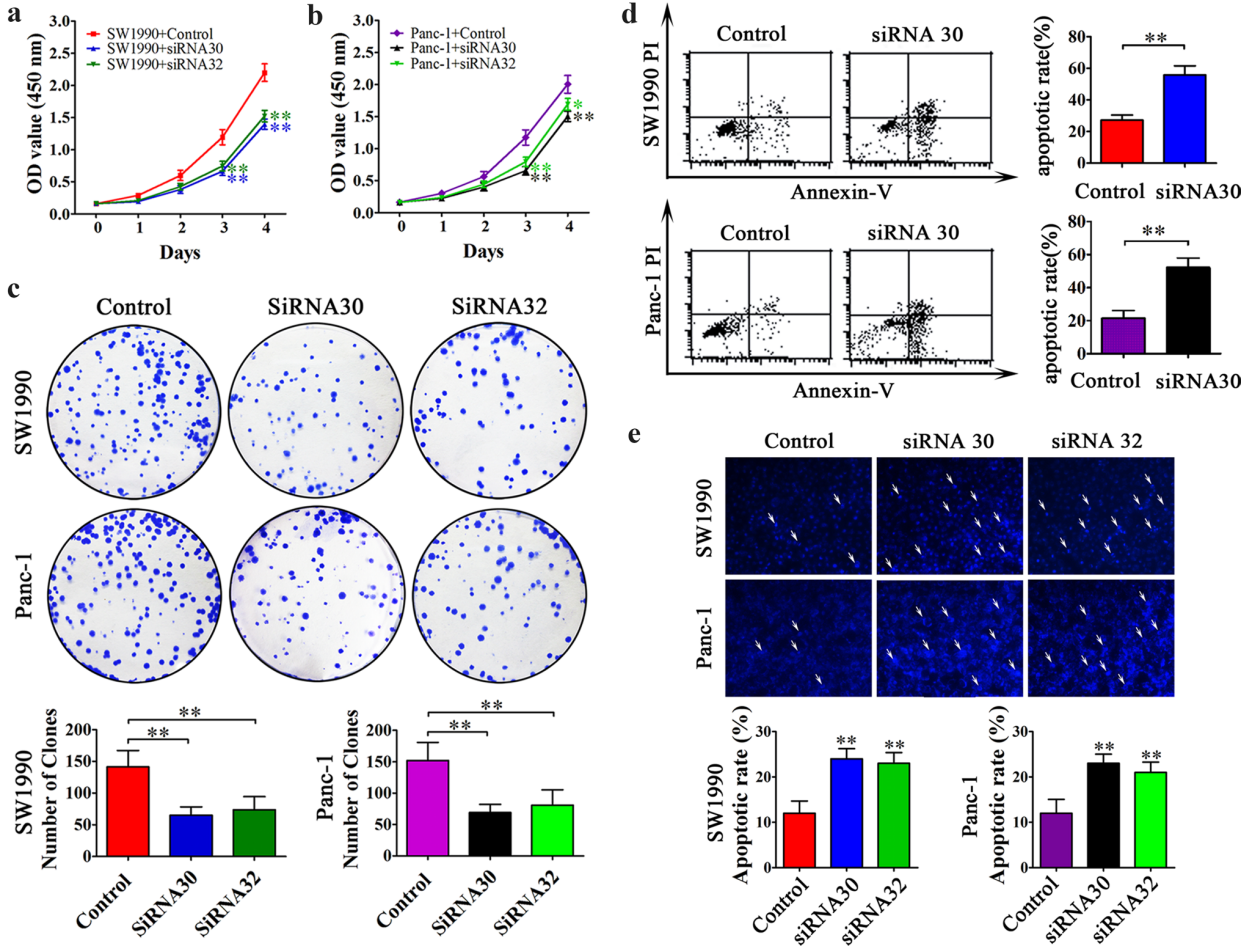
The role of ERas in PCCs proliferation and apoptosis in vitro. **a**, **b** SW1990 and Panc-1 cells transfected with ERas siRNA30, ERas siRNA32 or control siRNA were plated at 3 × 10^3^ cells/well in 96-well plates. CCK-8 assays were performed at 0, 24, 48, 72 and 96 h. The experiment was repeated three times and data are shown as mean ± SD (*P < 0.05 and **P < 0.01 vs. control). **c** After 24 h of siRNA30, siRNA32 and control transfection, 2 × 10^2^ cells were transferred into 6-well plates and plates were incubated for 2 weeks. Colonies were stained with crystal violet and photographed by an ordinary camera. Bar charts (bottom) show the number of colonies. The experiment was repeated three times and the significance was analyzed using the Student’s *t *test. Data are shown as mean ± SD (*P < 0.05 and ** *P* < 0.01 vs. control). **d** Apoptosis was evaluated in SW1990 and Panc-1 cells transfected with siRNA30 or control siRNA by Annexin V-PI staining and flow cytometry. The experiment was repeated three times and the representative histograms are shown. The apoptotic rates are shown as mean ± SD (*P < 0.05 and **P < 0.01 vs. control). **e** Apoptosis was evaluated in SW1990 and Panc-1 cells transfected as indicated and stained with Hoechst 33342 staining (original magnification × 200). The apoptotic rates of SW1990 and Panc-1 cells in five random fields were counted and the experiment was repeated three times independently. Data are shown as mean ± SD (**P < 0.01 vs. control)

We next examined the effect of ERas on pancreatic cancer pathogenesis. Our results showed that both SW1990 and Panc-1 exhibit high endogenous expression of ERas, and thus SW1990 and Panc-1 were used for subsequent functional assays. We downregulated the expression of ERas in the cell lines using two siRNAs (siRNA30 and siRNA32) and confirmed that the expression levels of ERas were markedly decreased in both SW1990 and Panc-1 cells after siRNA30 and siRNA32 transfection compared with cells transfected with control siRNA (Fig. S1). We next examined the role of ERas in PCCs proliferation. As shown in Fig. 2a, b, CCK-8 assays revealed a significant reduction of growth in both cell lines silenced for ERas gene expression compared with control cells. We also found that inhibition of ERas resulted in fewer and smaller colonies than the control groups in colony formation assays (Fig. 2c). These results demonstrate that downregulation of ERas significantly inhibited the proliferation and colony formation ability of PCCs.

### Downregulation of ERas promoted the apoptosis of PCCs

We further examined the effects of ERas on apoptosis in vitro using flow cytometry and Hoechst 33342. Flow cytometry assays showed that the apoptotic rate was significantly increased in ERas siRNA30-transfected cells compared with control cells (55.78% ± 6.21% vs. 28.21% ± 3.89% in SW1990, 52.19% ± 5.77% vs. 21.34% ± 4.85% in Panc-1, respectively; *P* < 0.01) (Fig. 2d). ERas siRNA-transfected cells exhibited the typical morphologic features of apoptosis, such as condensed chromatin and DNA fragments, as shown by Hoechst 33,342 staining (Fig. 2e). We observed more condensed and fragmented nuclei in ERas siRNA-treated cells compared with the control groups (*P* < 0.01).

### ERas promoted PCC migration and invasion in vitro

**Fig. 3:**
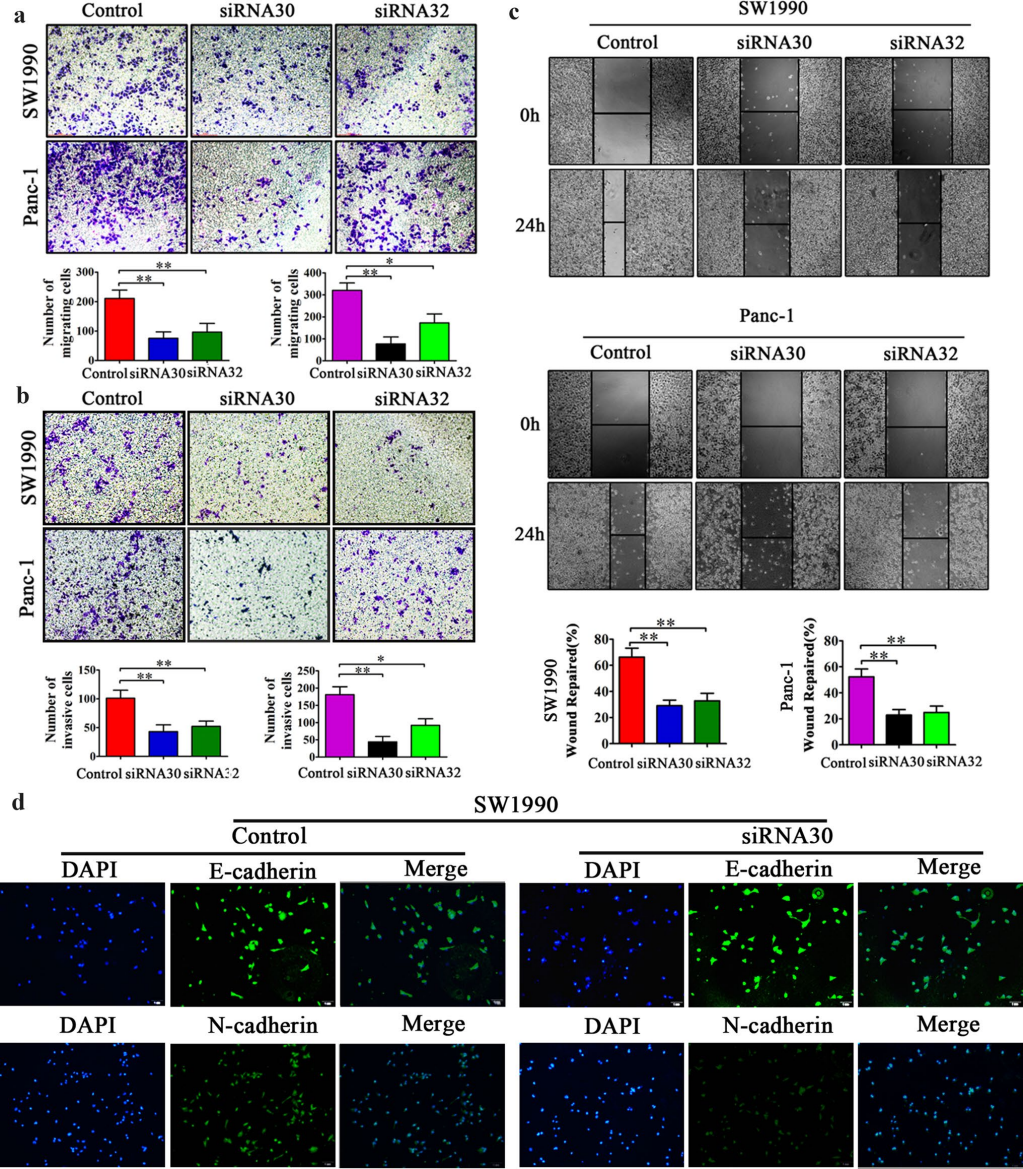
The role of ERas in SW1990 and Panc-1 cell migration. **a** After 24 h of siRNA30, siRNA32 and control siRNA transfection, 3 × 10^4^ cells were transferred to Transwell chambers and incubated for another 48 h. Cells were stained with crystal violet and observed by microscopy (× 50 magnification; Zeiss). The number of migrating cells in five random fields was counted using ImageJ software (× 100 magnification; Zeiss). The experiment was repeated three times and data are shown as mean ± SD (*P < 0.05, **P < 0.01 vs. control). **b** siRNA-transfected SW1990 (5 × 10^4^) and Panc-1 cells (5 × 10^4^) were resuspended in 200 µl serum-free medium and seeded into the upper chamber of Transwell chambers pre-coated with Matrigel. Cells were incubated for another 48 h and fixed with 4% paraformaldehyde containing 0.04% crystal violet for 20 min. Invasive cells were imaged and counted under a microscope in five random fields (× 100 magnification; Zeiss); The experiment was repeated three times and data are shown as mean ± SD (*P < 0.01, **P < 0.01 vs. control). **c** Migration activity was also measured by wound-healing assay. Migration distance was measured from five random fields captured at each indicated time point. The experiment was repeated three times and wound repair percentage of each cell line is shown in bar charts as mean ± SD (**P < 0.01 vs. control). (D) SW1990 transfected as indicated were analyzed by immunofluorescence using the indicated antibodies and captured images by Olympus BX-43 microscope. The experiment was repeated three times and the representative pictures are shown

We next used Transwell assay and wound-healing assay to ascertain the role of ERas in the migration and invasion of PCCs. The results showed that downregulation of ERas gene significantly inhibited SW1990 and Panc-1 cell migration and invasion compared with controls (Fig. 3a–c). These results suggest that ERas may play a key role in the metastasis of PCCs. As shown in Fig. 3d, the expression level of the mesenchymal cell marker N-cadherin decreased and the epithelial cell marker E-cadherin increased when ERas was downregulated by siRNA. Taken together, these data suggest that ERas promotes pancreatic cancer cell migration, invasion, and EMT.

### ERas promoted tumorigenicity and EMT of pancreatic cancer in vivo

**Fig. 4:**
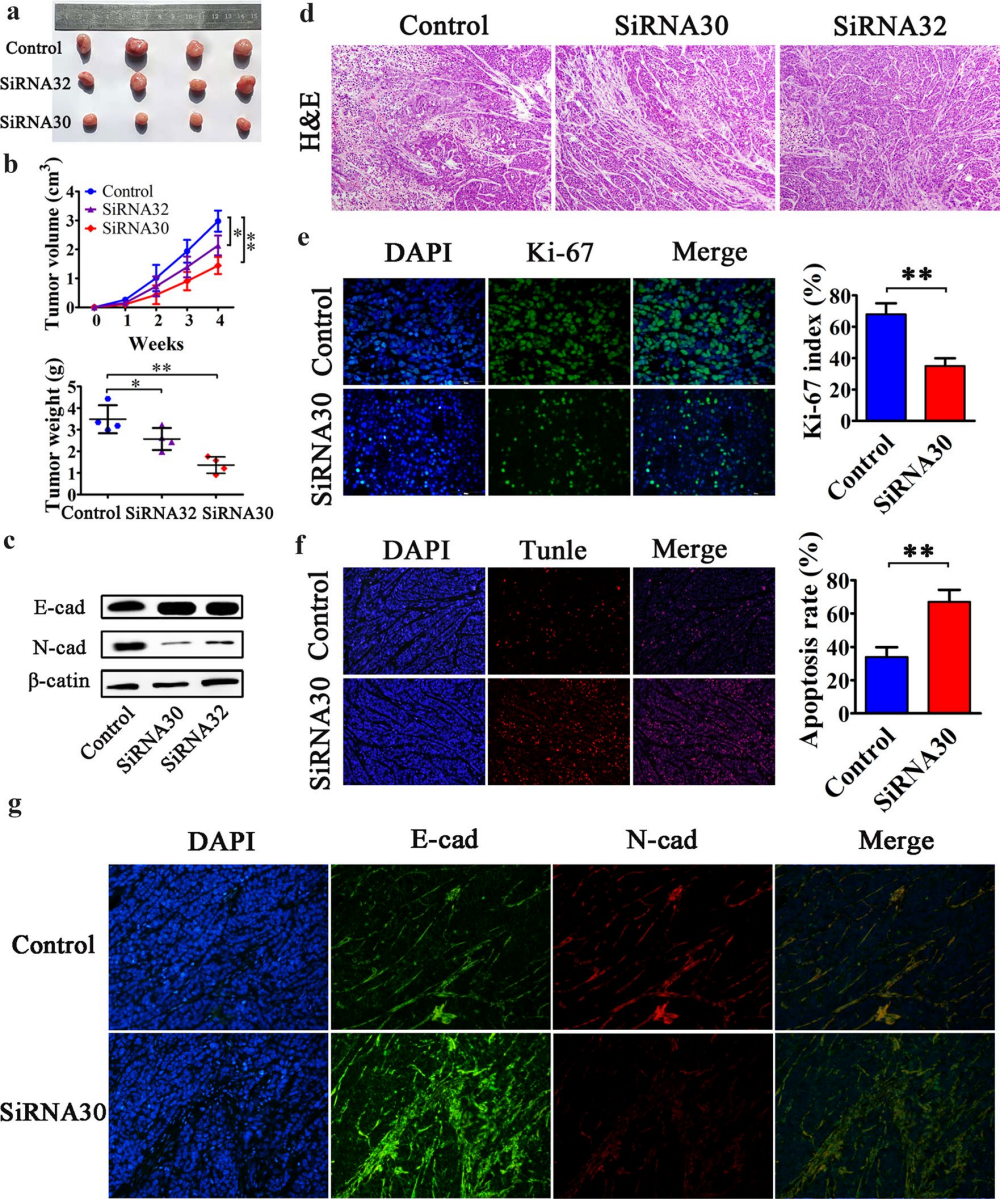
ERas promotes tumorigenicity in vivo and activates Erk/Akt signaling to regulate the activity of PCCs. **a** Control siRNA-, ERas siRNA30- and siRNA32-transfected SW1990 cells were injected into the right side of nude mice (*n* = 4/group). The mice were sacrificed after 4 weeks. ERas siRNA-transfected SW1990 cells exhibited slower growth compared with controls. **b** Reduced tumor volumes and weights were observed in xenografts derived from ERas siRNA-transfected SW1990 cells (*P < 0.01, **P < 0.01 vs. control). **c** SW1990 cells transfected with control siRNA, ERas siRNA30 and siRNA32 were harvested and E-cadherin, N-cadherin and β-actin expressions were determined by western blotting. **d** HE staining revealed the morphology of xenografts from the indicated groups. **e** Immunohistochemical staining showed that xenograft tumors derived from ERas siRNA30-expressing cells contained significantly fewer Ki-67-positive cells than those from the control group. The Ki-67 index was determined as the percentage of Ki-67-positive cells in 100 cells and the experiment was repeated three times independently. Data are shown in bar charts as mean ± SD (**P < 0.01, siRNA30 vs. control). **f** ERas significantly inhibited apoptosis in xenograft tumors of SW1990 cells as shown by TUNEL assay. The apoptosis rate was counted in five random fields and the experiment was repeated three times. Data are shown as mean ± SD (**P < 0.01, siRNA30 vs. control). **g** The expression of E-cadherin and N-cadherin in xenograft tumors were analyzed by immunofluorescence

We then explored the role of ERas in pancreatic cancer tumor progression in vivo. ERas downregulated SW1990 cells or control siRNA-treated SW1990 cells were subcutaneously injected into nude mice. After 4 weeks, the mice were killed, and the xenograft tumors were extracted (Fig. 4a). Decreased tumor volumes and weights were found in the ERas downregulation group compared with the control group (Fig. 4b). The expression of E-cadherin, N-cadherin and β-actin were determined by western blotting in siRNA-transfected subcutaneous tumors. We observed that N-cadherin expression was decreased and E-cadherin expression was increased in the siRNA30 and siRNA32 groups compared with controls (Fig. 4c). H&E staining showed the morphology of xenotransplanted tumor (Fig. 4d). Immunohistochemical staining revealed significantly fewer Ki-67-positive proliferative cells in xenograft tumors derived from cells transfected with ERas siRNA30 than in the control group (*P* < 0.01) (Fig. 4e). We also performed TUNEL assays to investigate the effects of ERas on cell apoptosis in vivo and observed that downregulation of ERas resulted in increased apoptosis in xenograft tumors of SW1990 cells (*P* < 0.01) (Fig. 4f). Besides, immunofluorescence staining also showed that N-cadherin expression was decreased and E-cadherin expression was increased in ERas downregulation group (Fig. 4g). Together these data indicate that ERas promotes pancreatic cancer tumor formation and EMT in vivo. Thus, ERas may act as a novel tumor-promoting factor and play a critical role in pancreatic cancer development.

### Regulation of PCCs activity by ERas is significantly associated with Erk/Akt signaling pathways

**Fig. 5:**
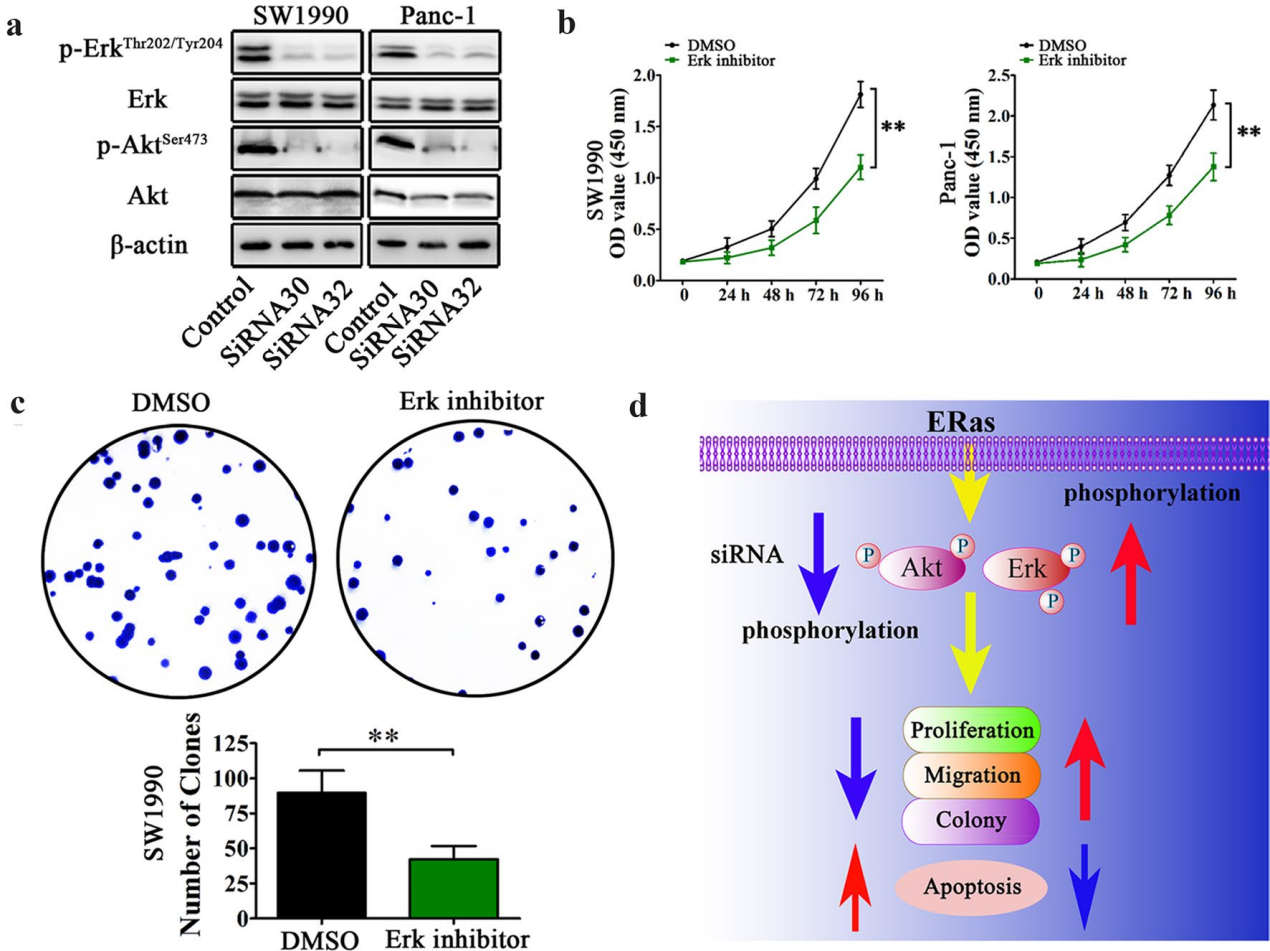
ERas activates Erk/Akt signaling to regulate the activity of PCCs. **a** Immunoblotting of phospho-Erk (Thr^202^/Tyr^204^) and phospho-Akt (Ser^473^) levels in SW1990 and Panc-1 cells treated with siRNA. ERas levels were normalized to those of β-actin. **b** Cell growth was inhibited by Erk inhibitor (SCH772984). **c** Colony formation ability was inhibited by Erk inhibitor (SCH772984) in SW1990 cells. **d** ERas activates Erk/Akt signaling to regulate the activity of pancreatic cancer cells

To ascertain the molecular mechanisms by which ERas promotes pancreatic cancer progression, we examined several signaling transduction pathways that might be crucial in tumorigenesis and Erk and Akt were focused on in the following experiments. As shown in Fig. 5a, the expression of phosphorylated Erk^Thr202/Tyr204^ and Akt^Ser473^ were reduced in the ERas-silenced cells compared with the control groups. We thus examined the role of Erk in pancreatic cell function using the Erk inhibitor SCH772984 and found the proliferation and colony formation of PCCs were inhibited by SCH772984 (Fig. 5b, c). These results suggest that ERas may regulate the proliferation, migration and colony formation of PCCs by regulating Erk-Akt signaling (Fig. 5d). These data also strongly suggest that Erk-Akt signaling is significantly associated with pancreatic cancer progression.

## Discussion

Pancreatic cancer is the most lethal malignancy in humans with aggressive local invasion and early metastasis [23,24] and an incidence that has increased over the last decades [25]. The Ras family plays a critical role in carcinogenesis and the development of pancreatic cancer. Growing evidence has supported the critical function of the Ras gene on the pathologic states of pancreatic cancer [26]. However, the expression and function of ERas, a member of the Ras family, in pancreatic cancer have been unknown.

ERas, previously named Hrasp, is located on the X chromosome and has a single open reading frame encoding a polypeptide with 76% identity to mouse ERas [20]. Previous studies have shown that ERas is pivotal in the occurrence and development of various malignant tumors, especially gastric cancer [19,20,27]. A previous study showed that ERas was strongly expressed in 142 gastric cancer tissues and closely related to liver or lymph node metastases [20]. In our study, we revealed that ERas is highly expressed in pancreatic cancer and exerted tumor-promoting effects. CCK-8, colony formation assays and Transwell assays revealed that ERas regulates PCC proliferation, colony formation, migration, invasion and metastasis activities as well as inhibits apoptosis in vitro. Importantly, subcutaneous tumor growth was inhibited in xenografts derived from cells with ERas knockdown. Ki-67 immunohistochemical staining demonstrated significantly fewer proliferative cells in ERas knockdown xenograft tumors, indicating ERas enhances the proliferation of PCCs in tumors. TUNEL assays also showed increased apoptotic cells in ERas knockdown xenograft tumors, suggesting ERas inhibits the apoptosis of tumor cells in vivo. Expression level of the mesenchymal cell marker N-cadherin was also decreased when ERas was downregulated by siRNA in vitro and in vivo. It is worth mentioning that we also found ERas was involve in the distant metastases, suggesting that ERas may serve as a potential diagnostic marker in pancreatic cancer.

Chemotherapy plays an important role in the comprehensive treatment of pancreatic cancer. However, resistance to chemotherapy is a big hurdle to treat this disease [28,29]. Thus, the determining mechanisms of pancreatic cancer may identify new alternate targets for treatment. The Akt pathway plays a critical role in the proliferation and activity of tumor cells. ERas may exert its function in pancreatic cancer by activating and regulating various downstream signaling transduction pathways. We hypothesized that ERas might modulate Erk and Akt signal transduction pathways to induce the cellular condition. Activation of signaling cascades by ERas may significantly enhance pancreatic cancer cells proliferation, migration, and colony formation ability and inhibit apoptosis. We found that inhibition of ERas expression by siRNA reduced the expression of Erk and Akt. In addition, inhibition of the Erk pathway using the Erk inhibitor SCH772984 resulted in greatly decreased proliferation and colony formation of pancreatic cancer cells. Together, these findings suggest that ERas may potentiate the activities of pancreatic cancer cells through the Erk and Akt pathway, suggesting that these pathways may represent a suitable target for the therapeutic treatment of pancreatic cancer.

In conclusion, we confirmed that ERas was overexpressed in pancreatic cancer tissues and PCCs. In addition, we revealed the biological role and potential signal pathways of ERas in pancreatic cancer progression. ERas was activated in pancreatic cancer cells, where ERas plays a critical role in PCCs survival and EMT. Our results suggest that chemotherapy strategies using low-dose Erk inhibitor drugs might provide a beneficial therapeutic approach for the clinical application of pancreatic cancer.

## Electronic supplementary material

Below is the link to the electronic supplementary material.

Supplementary file1 (DOCX 69 kb)
