# Supplementary material for: ERas regulates cell proliferation and epithelial–mesenchymal transition by affecting Erk/Akt signaling pathway in pancreatic cancer
Source: Hum Cell. 2020 Jul 22;33(4):1186–96. doi: 10.1007/s13577-020-00401-2 (PMC7505876; doi:10.1007/s13577-020-00401-2)
Supplement: Supplementary file 1 — Supplementary file1 (DOCX 69 kb) [file 13577_2020_401_MOESM1_ESM.docx]

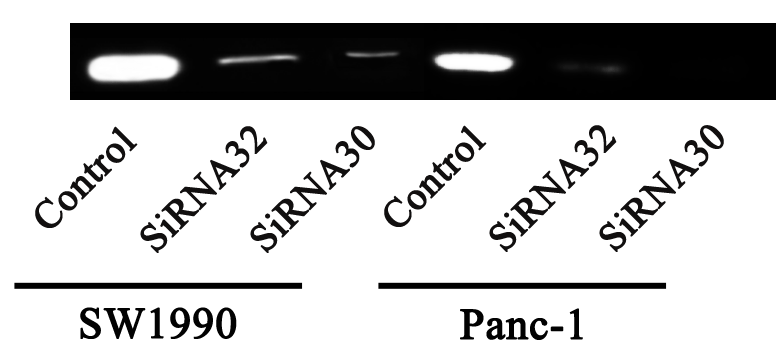


Fig. S1 ERas expression was markedly decreased in SW1990 and Panc-1 cells after siRNA30 and siRNA32 transfection compared with cells transfected with control siRNA.
